# Author Correction: Single-cell analysis of developing and azoospermia human testicles reveals central role of Sertoli cells

**DOI:** 10.1038/s41467-021-24242-1

**Published:** 2021-06-18

**Authors:** LiangYu Zhao, ChenCheng Yao, XiaoYu Xing, Tao Jing, Peng Li, ZiJue Zhu, Chao Yang, Jing Zhai, RuHui Tian, HuiXing Chen, JiaQiang Luo, NaChuan Liu, ZhiWen Deng, XiaoHan Lin, Na Li, Jing Fang, Jie Sun, ChenChen Wang, Zhi Zhou, Zheng Li

**Affiliations:** 1 Department of Andrology, the Center for Men’s Health, Urologic Medical Center, Shanghai Key Laboratory of Reproductive Medicine, Shanghai General Hospital, Shanghai Jiao Tong University School of Medicine, Shanghai, 200080 China; 2 School of Life Science and Technology, ShanghaiTech University, Shanghai, 201210 China; 3 Department of Urology, Shanghai Children’s Medical Center, School of Medicine, Shanghai Jiao Tong University, Shanghai, 200120 China; 4 Department of Andrology, the Affiliated Hospital of Qingdao University, Qingdao, 266000 Shandong China; 5 Shanghai Advanced Research Institute, Stem Cell and Reproductive Biology Laboratory, Chinese Academy of Sciences, Shanghai, 201210 China

**Keywords:** RNA sequencing, Stem-cell niche, Infertility, Urogenital reproductive disorders, Testis

Correction to: *Nature Communications* 10.1038/s41467-020-19414-4, published online 10 November 2020.

The original version of this Article contained an error in figure legend 2f, which incorrectly read ‘(f) Immunofluorescence co-staining of GATA4 (green) with JUN (red, upper panel), ENO1 (red, middle panel), and DEFB119 (red, lower panel) in human testicular paraffin sections at three ages..’ The correct version states ‘(f) Immunofluorescence co-staining of GATA4 (red) with JUN (green, upper panel), ENO1 (green, middle panel), and DEFB119 (green, lower panel) in human testicular paraffin sections at three ages.’. This has been corrected in both the PDF and HTML versions of the Article.

