# Supplementary material for: Single-cell analysis of developing and azoospermia human testicles reveals central role of Sertoli cells
Source: Nat Commun. 2020 Nov 10;11:5683. doi: 10.1038/s41467-020-19414-4 (PMC7655944; doi:10.1038/s41467-020-19414-4)
Supplement: Supplementary file 1 — Supplementary Information [file 41467_2020_19414_MOESM1_ESM.pdf]

Single-cell analysis of developing and azoospermia human testicles reveals central role of Sertoli cells

Liangyu Zhao et al.

Supplementary information

a

Sample ID	Group	Tech of single-cell capture	Total Cell Number	Sertoli Cell Number
LZ011	2 years	10X Genomics	7896	4793
LZ009	5 years	10X Genomics	4239	1744
LZ005	8 years	10X Genomics	7515	2046
LZ008	11 years	10X Genomics	5253	631
LZ016	17 years	10X Genomics	5007	834
LZ003/LZ007	23/28 years (OA)	10X Genomics	6342/8840	211/856
LZ013/LZ014/LZ015	25/28/31 years (OA)	BD Rhapsody	3197/3467/3530	408/27/89
LZ002	31 years (AZFa Microdeletions)	10X Genomics	5203	435
LZ004/LZ010/LZ012	26/27/29 years (Klinefelter's Syndrome)	10X Genomics	2743/2740/2244	117/115/30
LZ017/LZ018/LZ019	32/26/31 years (NOA)	BD Rhapsody	7402/6526/6579	992/682/974

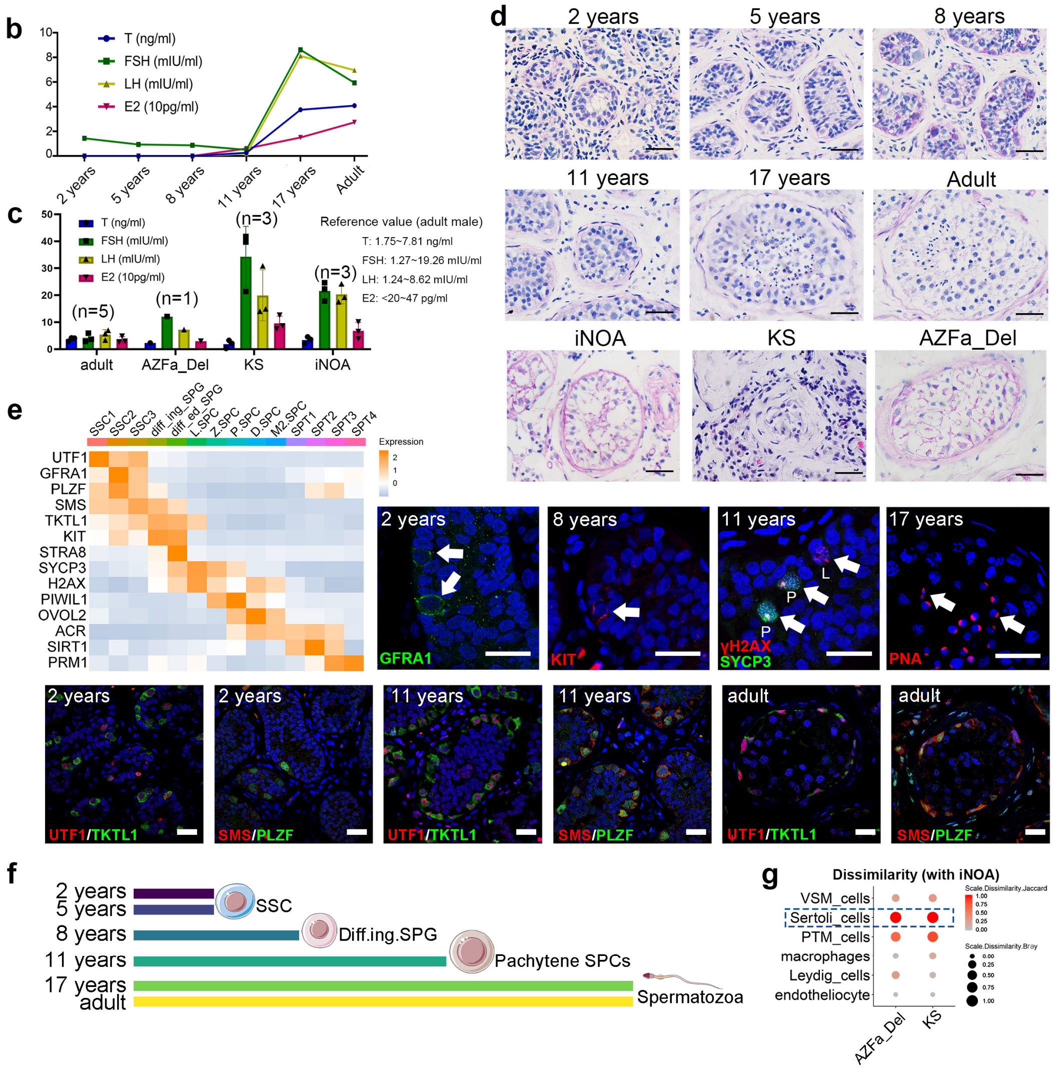

Supplementary Figure 1. **Clinical and histological information of enrolled samples.**

(a) Clinical information of enrolled samples in this study.

(b, c) Sex hormone levels of normal people at each age and three types of NOA patients. The sex hormone levels of adults are presented as the mean of 5 adult samples. T, testosterone; FSH, follicle-stimulating hormone; LH, luteinizing hormone; E2, estradiol. Data are presented as mean values ± SD.

(d) Periodic acid–Schiff (PAS) staining of each sample enrolled in this study illustrated the normal maturation and pathological changes in histological morphology with age.

(e, f) Heatmap and immunofluorescence staining for UTF1 (SSC marker), GFRA1 (SSC marker), PLZF (SSC marker), SMS (SSC and SPG marker), KIT (SPG marker), TKTL1 (SPG and early SPC marker) and PNA (spermatid marker) and double staining of SYCP3 and γH2AX (SPC markers) in testicular paraffin sections of each age, illustrating the spermatogenic maturity in each sample. The scale bar represents 20 μm. Some elements in Supplementary Figure 1f were downloaded from Servier Medical Art repository.

(g) Dissimilarity of somatic cells between iNOA and AZFa_Del or KS are shown on bubble diagram. The gradient of bubbles sizes indicates low to high scaled Bary value, and the gradient of red indicates low to high scaled Jaccard values.

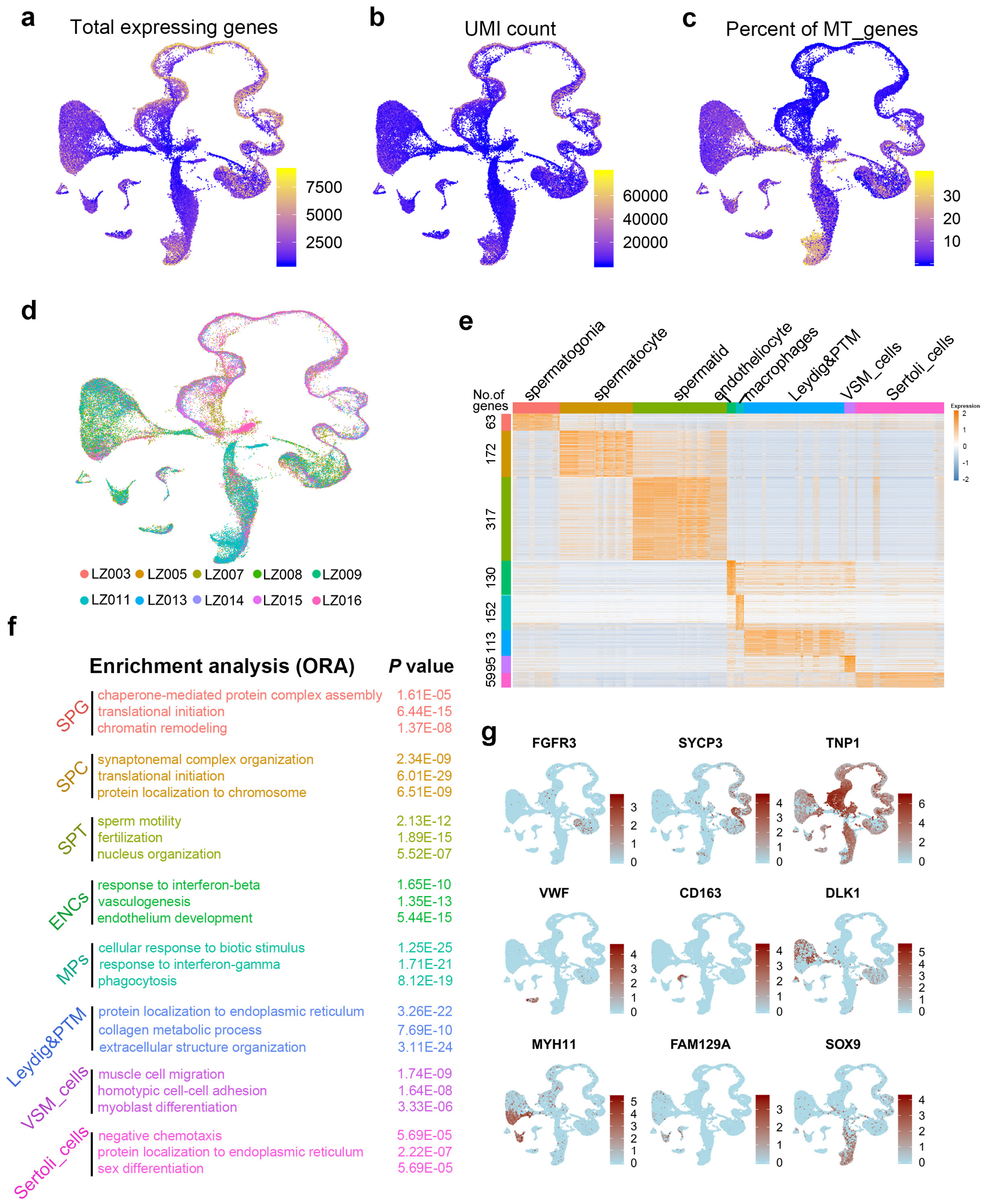

Supplementary Figure 2. Quality control of single-cell RNA-seq datasets and identification of nine clusters of normal developing testicular cells.

(a–c) Single-cell RNA-seq quality information, including (a) total count of expressed genes, (b) UMI counts, and (c) percentage of mitochondrial gene expression, is projected on the UMAP plot.

(d) UMAP plots of all 10 healthy human testicular cell samples. Each sample is labeled with a different color.

(e) Heatmap showing the DEGs of each cluster. DEG counts are shown on the left of the color bar of the cell type annotation.

(f) Enriched GO terms and P-values of each cell cluster are indicated by eight distinct colors. (g) Expression patterns of the following markers for each cluster are projected on the UMAP plot: FGFR3 (spermatogonia), SYCP3 (spermatocytes), TNP1 (spermatids), VWF (endotheliocytes), CD63 (macrophages), DLK and MYH11 (MIX cells), FAM129A (vascular smooth muscle cells), and SOX9 (Sertoli cells). A gradient of light blue to dark red indicates low to high expression levels.

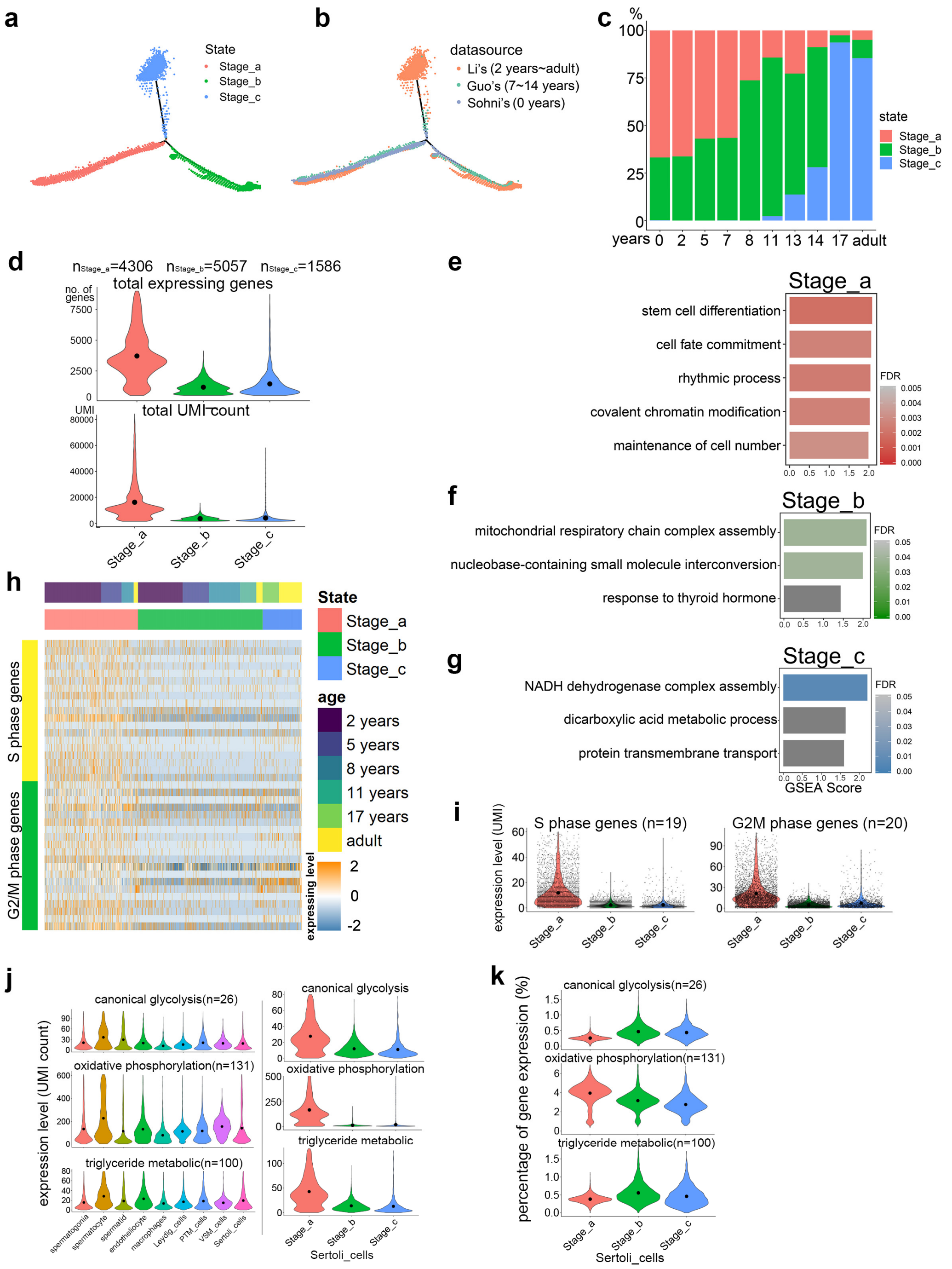

Supplementary Figure 3. Characteristics related to the cell cycle, energy metabolism, and

biological processes of each stage during Sertoli cell development.

(a, b) Three-stage pattern of Sertoli cells which are from three different laboratories is shown on pseudotime trajectory plot.

(c) Bar plot shows the proportion of Sertoli cells at each stage (Stage_a, red; Stage_b, green; Stage_c, blue) in each age group.

(d) Violin plot of overall transcriptional levels of Sertoli cells at each stage.

(e–g) Pathways and biological process terms of GSEA are shown as barplot. The GSEA score is presented on the x-axis, and a gradient of three colors indicates low to high FDR values of the three stages.

(h) Heatmap of cell cycle-specific genes in Sertoli cells at each stage.

(i) Violin plot of the expression levels of S phase and G2M phase specific genes in three stages of Sertoli cells.

(j) Violin plot of the expression levels of energy metabolism-related genes of nine types of testicular cells (left panel) and Sertoli cells at each stage (right panel).

(k) Violin plot of the percentage of energy metabolism-related genes of Sertoli cells at each stage.

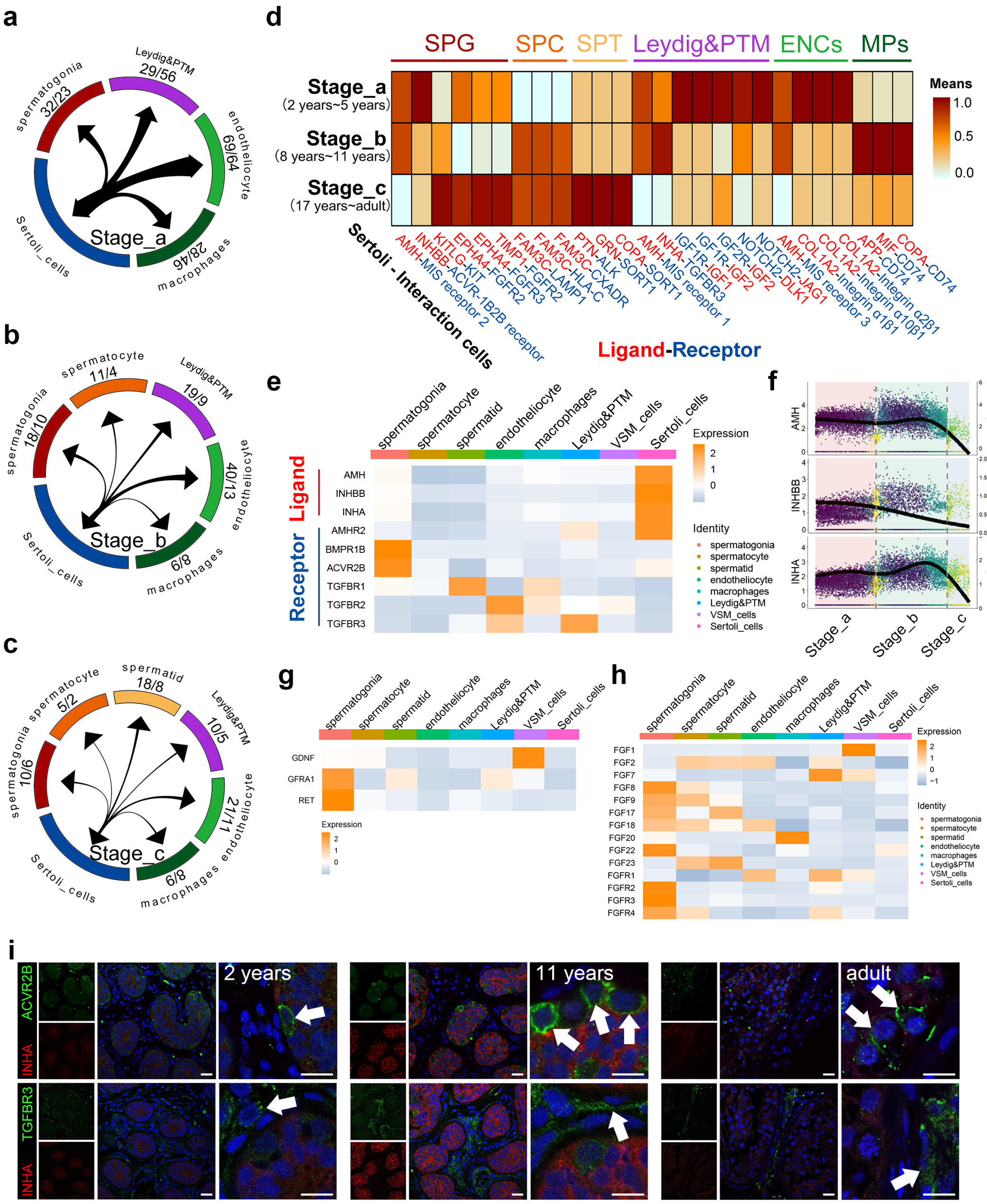

Supplementary Figure 4. Dynamic changes of the interactions between Sertoli cells and other testicular cells.

(a–c) Loop graph shows the number of ligands–receptors interactions between Sertoli cells at each stage and other testicular cells. The numbers of ligands/receptors from Sertoli cells are shown under the cell type annotation.

(d) Heatmap shows the matching strength of the interaction between Sertoli cells and other testicular cells.

(e, f) Heatmap shows the spatial expression pattern of ligands and receptors of the TGF-β signaling pathway (e), point plot showing the temporal expression patterns of ligands secreted by Sertoli cells (f). The pseudotime is presented on the x-axis, and mean expression level is presented by the trend line.

(g, h) Heatmap shows the spatial expression pattern of ligands and receptors of the GDNF-GFRA1/RET and FGFs-FGFRs signaling.

(i) Immunofluorescence co-staining of INHA (red) with ACVR2B (green, upper panel) and TGFBR3 (green, lower panel) in human testicular paraffin sections at three ages. The scale bar represents 15 μm.

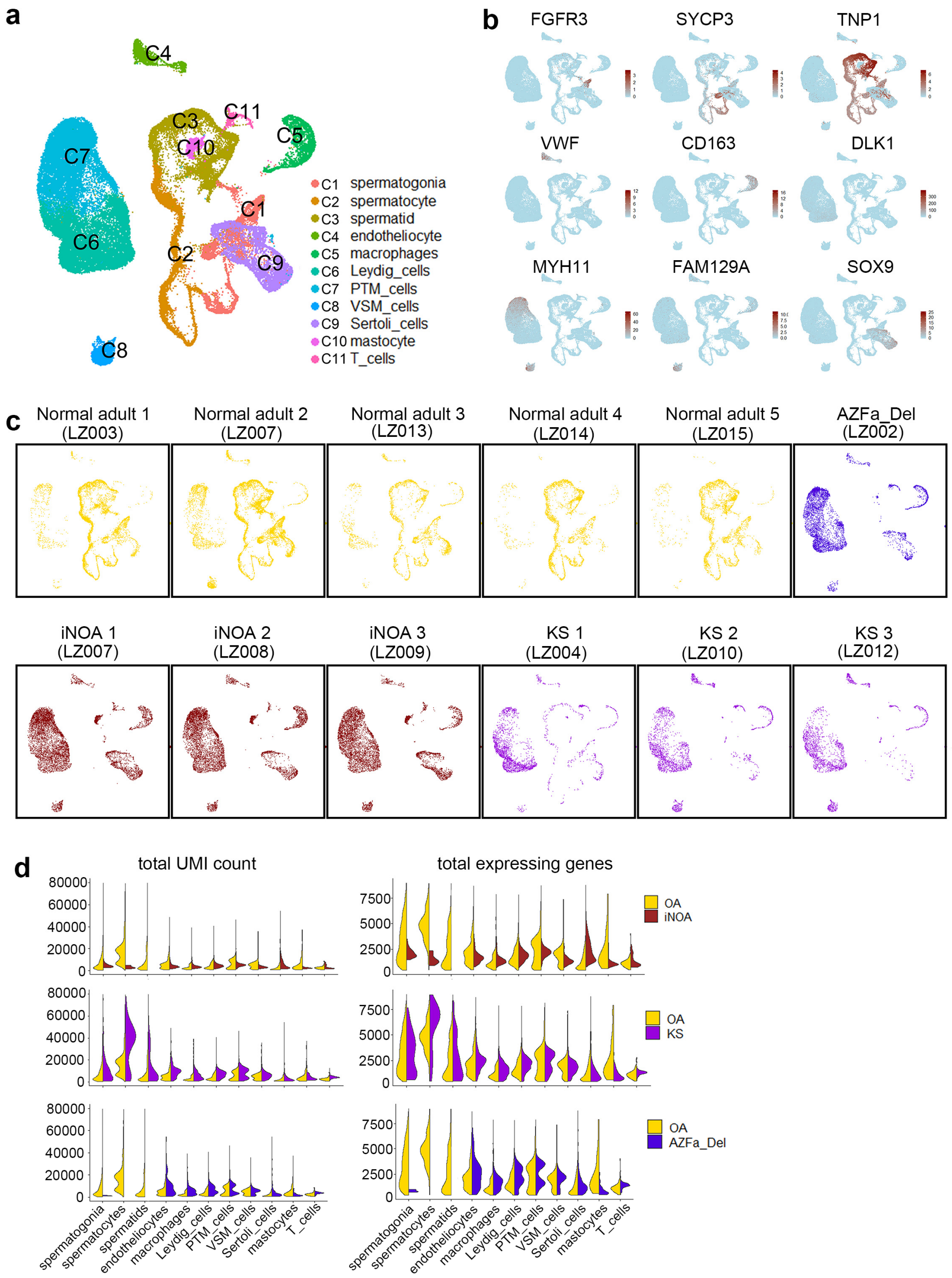

Supplementary Figure 5. The heterogeneity among different types of NOA and the repeatability with samples of the same type of NOA.

(a) UMAP plots of 10 healthy samples combined with three types of NOA.

(b) Expression patterns of the following markers for each cluster are projected on the UMAP plot: FGFR3 (spermatogonia), SYCP3 (spermatocytes), TNP1 (spermatids), VWF

(endotheliocytes), CD63 (macrophages), DLK and MYH11 (MIX cells), FAM129A (vascular smooth muscle cells), and SOX9 (Sertoli cells). A gradient of light blue to dark red indicates low to high expression levels.

(c) UMAP plot of each isolated sample. Healthy and different NOA cells are colored differently.

(d) Violin plot of overall transcriptional level of different cell clusters. Healthy cells and different NOA clusters are split in different columns.

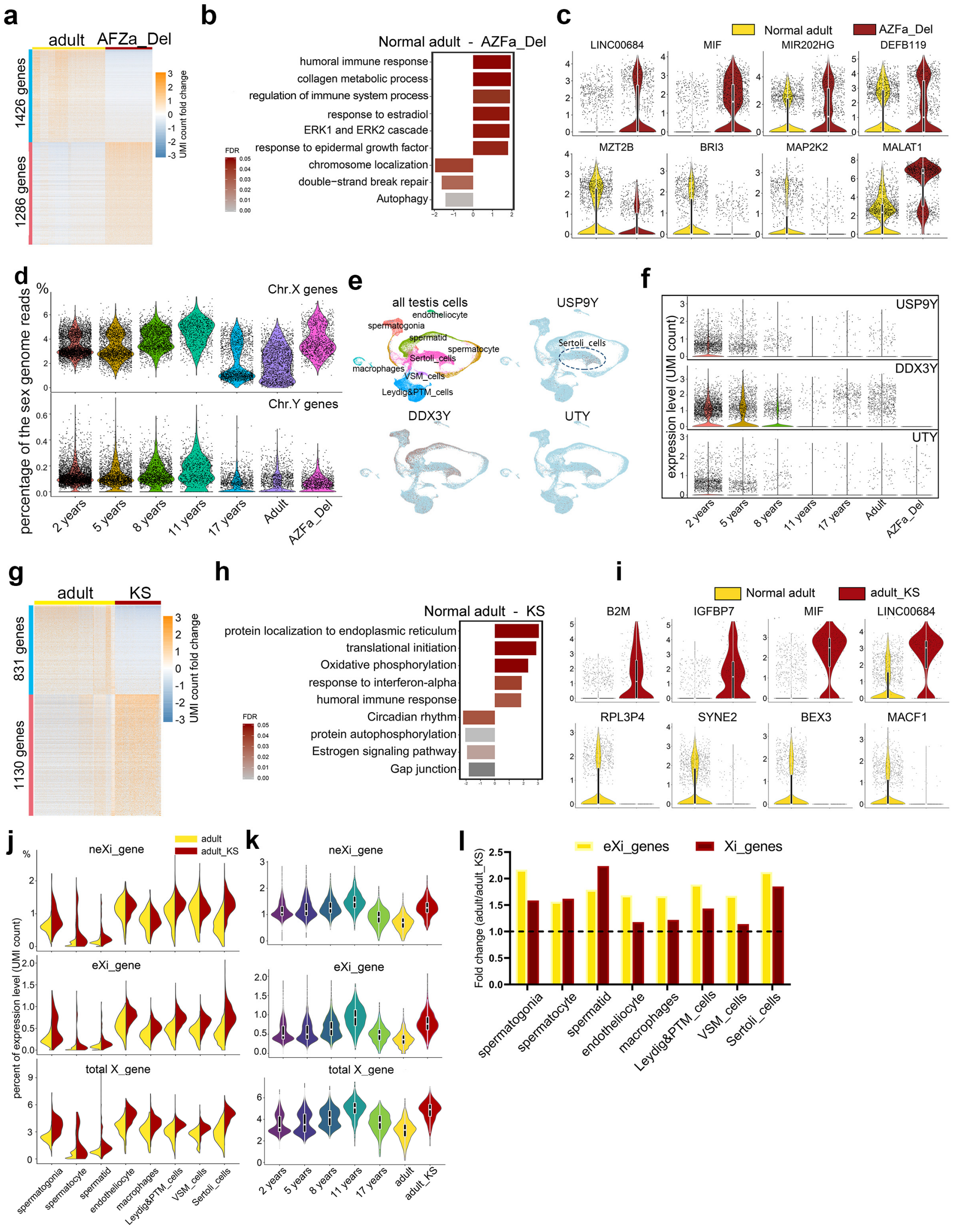

Supplementary Figure 6. Abnormal expression patterns of Sertoli cells in AZFa_Del and KS.

(a) Heatmap of DEGs between healthy adult Sertoli cells and AZFa_Del Sertoli cells.

(b) Violin plot of the expression levels of the top four positive and negative DEGs in AZFa_Del Sertoli cells.

(c) GSEA terms enriched and decreased in AZFa_Del Sertoli cells, shown as barplot. The GSEA score is presented on the x-axis, and the gradient of red indicates low to high FDR values.

(d) Violin plot of the expression levels of sex chromosomal genes at in normal Sertoli cells (six different ages) and AZFa_Del Sertoli cells.

(e) UMAP plot shows the expression patterns of three genes in the AZFa region.

(f) Violin plot of the expression levels of three AZFa genes in normal Sertoli cells (six different ages) and AZFa_Del Sertoli cells.

(g) Heatmap of DEGs between normal adult Sertoli cells and KS Sertoli cells.

(h) Violin plot of the expression levels of the top four positive and negative DEGs in KS Sertoli cells.

(i) GSEA terms enriched and decreased in KS Sertoli cells, shown as barplot. The GSEA score is presented on the x-axis, and the gradient of red indicates low to high FDR values.

(j, k) Violin plot of the expression level of eXi and neXi genes (J) in eight types of testicular cells and (K) in normal (six different ages) and AZFa_Del Sertoli cells.

(l) Barplot showing the ratio of the expression levels of eXi (yellow) and neXi (dark red) expression levels between healthy adults and KS patients.

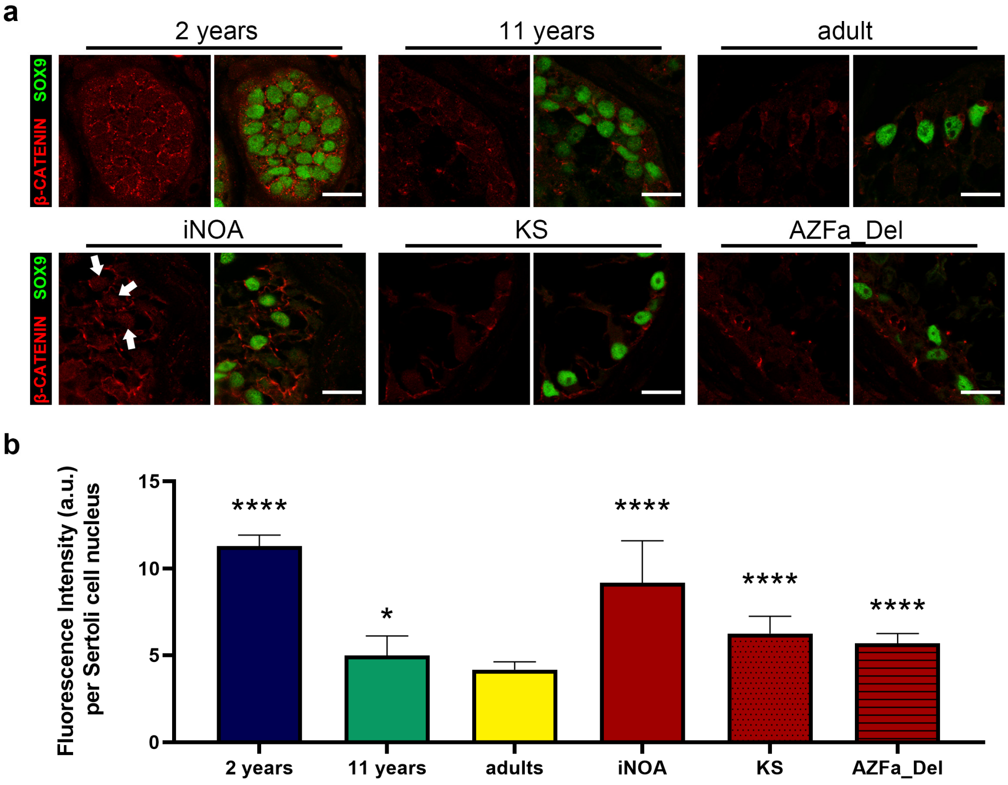

Supplementary Figure 7. Different activation level of Wnt/β-catenin pathway in normal development and NOA testis.

(a) Immunofluorescence co-staining of β-catenin (red) with SOX9 (green) in human testicular paraffin sections at three ages and three types of NOA. The long arrow marks high expression

level of β-catenin in nucleus of iNOA Sertoli cells. The scale bar represents 20 μm.

(b) The expression level of nucleus located β-catenin is shown as histogram. Data shown as mean fold-change (±SD) relative to fluorescence intensity per Sertoli cell nucleus in 6 type of testicular samples. Statistical analysis between normal adult and other groups made by non-parametric test with Mann-Whitney test; two-tailed. *p<0.05, ****p<0.0001 (comparing with normal adult).

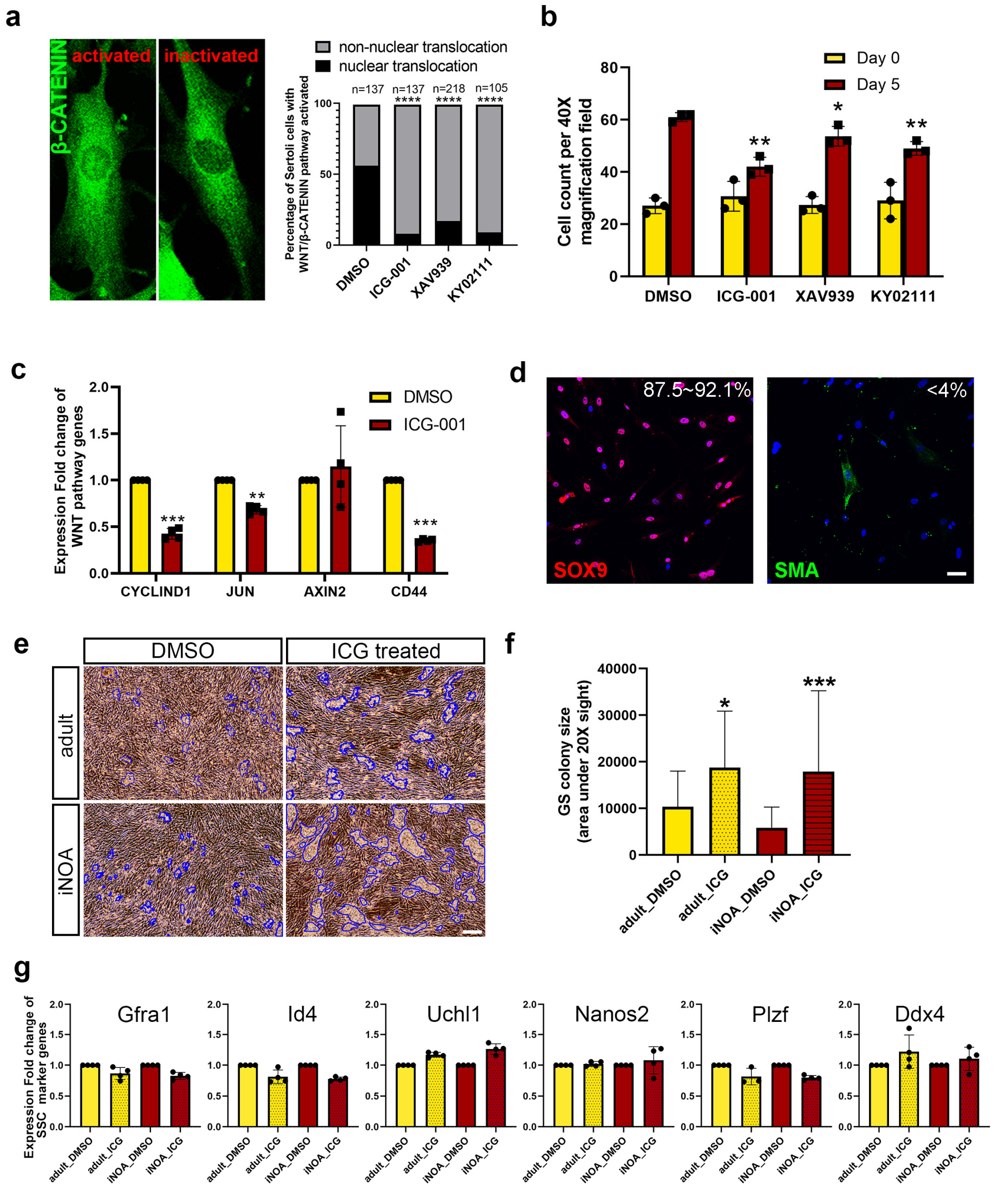

Supplementary Figure 8. Inhibition of Wnt/β-catenin pathway in Sertoli cells.

(a) Immunocytochemistry staining of β-catenin (green) in Sertoli cells shows the activated and inactivated state of Wnt/β-catenin pathway. The right panel showed the percentage of Sertoli cells with β-catenin nucleus translocation after treated with Wnt/β-catenin pathway inhibitors. Data shown as mean ± SD from 3 biological independent samples. Statistical analysis between DMSO and WNT inhibitors inducing group made by chi-square test. *p<0.05, ****p<0.0001

(comparing with normal adult).

(b) Cell count of Sertoli cells treated with DMSO or three types of Wnt/β-catenin pathway inhibitors for five days in vitro. Data shown as mean ± SD from 3 biological independent samples. Statistical analysis between DMSO and WNT inhibitors in day 5th made by two-tailed, unpaired non-parametric test with Mann-Whitney test; the confidence interval is 95%. *p<0.05, ***p<0.001 (comparing with DMSO treated group).

(c) qPCR results shows the expression fold change of classical Wnt/β-catenin pathway target genes in cultured iNOA and normal adult Sertoli cells with or without ICG treatment. The gene expression levels of normal adult Sertoli cells without ICG treatment (DMSO treated) were used as the baseline values. Data shown as mean ± SD from 4 biological independent samples. Statistical analysis made by two-tailed, unpaired Student's t-test; the confidence interval is 95%. **p<0.01, ***p<0.001 (comparing with DMSO treated group).

(d) Immunocytochemistry staining of SOX9 (red) and SMA (green) in Sertoli cells with ICG treatment for 14 days, and the percentage of SOX/SMA positive cells was noted according to three independent fields at 20X magnification.

(e, f) The colony size of cultured GS cells using iNOA and normal adult Sertoli cells with or without ICG treatment as feeder cells. The colonies are surrounded by blue lines. (e). The statistics of colony size is shown as histogram (f). Data shown as mean ± SD from 3 biological independent samples. Statistical analysis between DMSO and ICG treatment group made by two-tailed, unpaired non-parametric test with Mann-Whitney test; the confidence interval is 95%. *p<0.05, ***p<0.001 (comparing with DMSO treated group).

(g) qPCR results shows the expression fold change of SSC markers in cultured GS cells using iNOA and normal adult Sertoli cells with or without ICG treatment as feeder cells. Data shown as mean ± SD from 4 biological independent samples. Statistical analysis made by two-tailed, unpaired Student's t-test; the confidence interval is 95%. *p<0.05, ***p<0.001 (The gene expression levels of GS cells cultured with DMSO treated Sertoli cells are used as the baseline values).
