# Supplementary material for: Single-cell analysis of developing and azoospermia human testicles reveals central role of Sertoli cells
Source: Nat Commun. 2020 Nov 10;11:5683. doi: 10.1038/s41467-020-19414-4 (PMC7655944; doi:10.1038/s41467-020-19414-4)
Supplement: Supplementary file 9 — Reporting Summary [file 41467_2020_19414_MOESM9_ESM.pdf]

nature research

Corresponding author(s): Zheng Li; Zhi Zhou; ChenChen Wang; Jie Sun

Last updated by author(s): Sep 29, 2020

Reporting Summary

Nature Research wishes to improve the reproducibility of the work that we publish. This form provides structure for consistency and transparency in reporting. For further information on Nature Research policies, see our Editorial Policies and the Editorial Policy Checklist.

Statistics

For all statistical analyses, confirm that the following items are present in the figure legend, table legend, main text, or Methods section.

n/a	Confirmed	
☐	☒	The exact sample size (*n*) for each experimental group/condition, given as a discrete number and unit of measurement
☐	☒	A statement on whether measurements were taken from distinct samples or whether the same sample was measured repeatedly
☐	☒	The statistical test(s) used AND whether they are one- or two-sided<br>*Only common tests should be described solely by name; describe more complex techniques in the Methods section.*
☐	☒	A description of all covariates tested
☐	☒	A description of any assumptions or corrections, such as tests of normality and adjustment for multiple comparisons
☐	☒	A full description of the statistical parameters including central tendency (e.g. means) or other basic estimates (e.g. regression coefficient) AND variation (e.g. standard deviation) or associated estimates of uncertainty (e.g. confidence intervals)
☐	☒	For null hypothesis testing, the test statistic (e.g. *F*, *t*, *r*) with confidence intervals, effect sizes, degrees of freedom and *P* value noted<br>*Give P values as exact values whenever suitable.*
☒	☐	For Bayesian analysis, information on the choice of priors and Markov chain Monte Carlo settings
☐	☒	For hierarchical and complex designs, identification of the appropriate level for tests and full reporting of outcomes
☒	☐	Estimates of effect sizes (e.g. Cohen's *d*, Pearson's *r*), indicating how they were calculated

Our web collection on statistics for biologists contains articles on many of the points above.

Software and code

Policy information about availability of computer code

Data collection	The software used for collecting qRT-PCR data was Bio-Rad CFX manager 3.1. Images were captured with an OLYMPUS IX83 confocal microscope
Data analysis	R version 3.6.0 (2019-04-26)<br>Version of R packages:<br>Seurat_3.0.0<br>gplots_3.0.1<br>dplyr_0.7.4<br>Biobase_2.38.0<br>ggplot2_2.2.1<br>monocle_2.6.1<br>DDRTree_0.1.5<br>stringr_1.2.0<br>ggpubr_0.1.6<br>Ssmarina 1.0.1<br>Vegan 2.5.6<br><br>Other softwares:<br>Cell Ranger 2.2.0<br>ARACNe-AP 1.0.0<br>GraphPad Prism 8<br>Ingenuity Pathway Analysis, IPA 3.15

CellPhoneDB v2.0
WebGestalt 2019
Photoshop CS6

For manuscripts utilizing custom algorithms or software that are central to the research but not yet described in published literature, software must be made available to editors and reviewers. We strongly encourage code deposition in a community repository (e.g. GitHub). See the Nature Research guidelines for submitting code & software for further information.

Data

Policy information about availability of data

All manuscripts must include a data availability statement. This statement should provide the following information, where applicable:

- Accession codes, unique identifiers, or web links for publicly available datasets
- A list of figures that have associated raw data
- A description of any restrictions on data availability

The RNA-seq matrix data generated in this study has been deposited in NCBI with accession number GSE149512 (this study). Other sequencing datasets used can be found in NCBI GEO under accession numbers GSE134144 and GSE124263 (Guo's and Sohni's data for Figure S3a-c). The list of TFs can be obtained in Supplementary Table 7 of this study or from http://bioinfo.life.hust.edu.cn/AnimalTFDB/#!/. The annotation of gene function analysis can also be obtained in Supplementary Table 7 of this study or from http://amigo.geneontology.org/amigo/landing/. All other relevant data supporting the key findings of this study are available within the article and its Supplementary Information files or from the corresponding author upon reasonable request. A reporting summary for this Article is available as a Supplementary Information file. Raw data associated with each figure are provided in the Source Data table of this paper. Some elements in Figure 1a, 7 and Supplementary Figure 1f such as the images of germ cells and Sertoli cells were downloaded from Servier Medical Art repository. All code associated with this manuscript and the gene annotation list have been uploaded to GitHub (https://github.com/zlyingithub/17-testis-single-cell-R-github-data).

Field-specific reporting

Please select the one below that is the best fit for your research. If you are not sure, read the appropriate sections before making your selection.

☒ Life sciences ☐ Behavioural & social sciences ☐ Ecological, evolutionary & environmental sciences

For a reference copy of the document with all sections, see nature.com/documents/nr-reporting-summary-flat.pdf

Life sciences study design

All studies must disclose on these points even when the disclosure is negative.

Sample size	No statistical method was used to predetermine sample size. > 80,000 human testicular single-cell transcriptomes from 10 donors with normal spermatogenesis spanning the range from infant to adult and 7 NOA patients. This samples cover infant, childhood, puberty and adult during development. The samples of each adjacent age showed a gradual trend, so these samples are sufficient.
Data exclusions	No data were excluded.
Replication	Testicular single-cell transcriptomes from normal adult, Klinefelter's syndrome and iNOA patients were three biological replicates. Three samples within one same disease showed good repeatability (results showed in Fig S5C), no sample was excluded. The results of qPCR and supporting the culture of spermatogenic cells were repeated in three biological replicates and showed good repeatability.
Randomization	For cell culture and inducing experiments, primary Sertoli cells were obtained from 3 OA patient and 3 iNOA patients, these patients were not completely randomly selected, but they have similar clinical data, including age, testicular size, hormone levels, and etiology, so we thought these patients could represent the general features of these diseases. P1 primary Sertoli cells were digested and mix well, then divided into 4 wells randomly for functional experiment such as different WNT inhibitor inducing or CCK8 array.
Blinding	The type and ratio of testis cells were largely different between young and old donors and the bioinformatics analysis should be done according to their biological background thus unsuitable for blinding experiments. The results involved in this study were all objective indicators, such as the results of IHC fluorescence intensity, and QPCR. Statistical results were also obtained from multiple random fields, so the blindness did not affect the results of this study.

Reporting for specific materials, systems and methods

We require information from authors about some types of materials, experimental systems and methods used in many studies. Here, indicate whether each material, system or method listed is relevant to your study. If you are not sure if a list item applies to your research, read the appropriate section before selecting a response.

Materials & experimental systems

n/a	Involved in the study
	☒ Antibodies
	☒ Eukaryotic cell lines
☒	Palaeontology and archaeology
☒	Animals and other organisms
	☒ Human research participants
☒	Clinical data
☒	Dual use research of concern

Methods

n/a	Involved in the study
☒	ChIP-seq
☒	Flow cytometry
☒	MRI-based neuroimaging

Antibodies

Antibodies used

Goat monoclonal Human GFR alpha-1/GDNF R alpha-1 Antibody R&D AF714; IHC 1:50
Mouse Anti-UFT1 Antibody millipore MAB4337; IHC 1:200
Mouse Anti-UCHL1 Antibody BIO-RAD MCA4750GA; IHC 1:200
Rabbit Anti-TKTL1 Antibody Novus NBP1-86939; IHC 1:200
Rabbit Anti-PLZF Antibody Santa Cruz sc-22839; IHC 1:200
Mouse Anti-SPSY (SMS) Antibody Santa Cruz sc-376294; IHC 1:200
Rabbit monoclonal Anti-c-Kit antibody [YR145] Abcam Ab32363; IHC 1:50
Goat monoclonal Human SCP3/SYCP3 Antibody R&D AF3750 ; IHC 1:200
Mouse monoclonal Anti-phospho-Histone H2A.X (Ser139) Antibody Sigma 05-636-I; IHC 1:200
Rabbit monoclonal Anti-c-Jun antibody [E254] Abcam Ab32137; IHC 1:200
Mouse monoclonal Anti-Enolase Antibody (A-5) Santa Cruz sc-271384; IHC 1:200
Rabbit Polyclonal Anti-DEFB119 Antibody Atlas Antibodies HPA043059; IHC 1:500
Mouse monoclonal Anti-GATA-4 Antibody (G-4) Santa Cruz sc-25310; IHC 1:200
Rabbit monoclonal Anti-ZO-1 antibody (D6L1E) CST #13663; IHC 1:200
Mouse monoclonal Anti-EGR3 Antibody (A-7) Santa Cruz sc-390967; IHC 1:200
Rabbit Polyclonal Anti-HOPX Antibody Proteintech 11419; IHC 1:100
Mouse monoclonal Anti-HOPX (E-1) Antibody Santa Cruz; sc-398703; IHC 1:100
Rabbit Polyclonal Anti-INHA Antibody Atlas Antibodies HPA019141; IHC 1:200
Mouse monoclonal Human Activin RIIB Antibody R&D MAB3392; IHC 1:200
Mouse monoclonal Anti-TGFβ RIII (A4) Antibody Santa Cruz sc-74511; IHC 1:200
Rabbit Polyclonal SOX9 Antibody millipore AB5535; IHC 1:400
Rabbit monoclonal [SP171] SMA Antibody Abcam ab150301; IHC 1:200
Mouse monoclonal Anti-Active-β-Catenin (Anti-ABC) Antibody millipore 05-665; IHC 1:200
Mouse monoclonal Anti-β-Catenin Antibody Servicebio GB12015; ICC 1:200
Alexa Fluor 488 donkey anti-rabbit IgG Thermo Fisher Scientific A21206; RRID: AB_2535792 ; IHC 1:500
Alexa Fluor 555 donkey anti-rabbit IgG Thermo Fisher Scientific A31572; RRID: AB_10562716; IHC 1:500
Alexa Fluor 488 donkey anti-mouse IgG Thermo Fisher Scientific A21202; RRID: AB_141607; IHC 1:500
Alexa Fluor 555 donkey anti-mouse IgG Thermo Fisher Scientific A31570 RRID: AB_2313501; IHC 1:500
Alexa Fluor 488 donkey anti-goat IgG Thermo Fisher Scientific A21432 RRID: AB_10053826; IHC 1:500

Validation

Goat monoclonal Human GFR alpha-1/GDNF R alpha-1 Antibody R&D AF714; Suitable for IHC/ICC of human. Validated for detecting membrane of SSC in testis.
Mouse Anti-UFT1 Antibody millipore MAB4337; IHC 1:200; Suitable for IHC of human. Validated for detecting SSC in testis.
Mouse Anti-UCHL1 Antibody BIO-RAD MCA4750GA; IHC 1:200; Suitable for IHC/ICC of human and mouse. Validated for detecting cytoplasm of SSC in testis.
Rabbit Anti-TKTL1 Antibody Novus NBP1-86939; Suitable for WB, IHC of human. Validated for detecting cytoplasm of SPG in testis.
Rabbit Anti-PLZF Antibody Santa Cruz sc-22839; Suitable for WB, IHC/ICC of human and mouse. Validated for detecting nucleus of SSC in testis.
Mouse Anti-SPSY (SMS) Antibody Santa Cruz sc-376294; Suitable for IHC of human. Validated for detecting nucleus of SSC in testis.
Rabbit monoclonal Anti-c-Kit antibody [YR145] Abcam Ab32363; Suitable for IHC/ICC of human. Validated for detecting membrane of SPG in testis.
Goat monoclonal Human SCP3/SYCP3 Antibody R&D AF3750 ; Suitable for IHC/ICC of human and mouse. Validated for detecting nucleus of SPC in testis.
Mouse monoclonal Anti-phospho-Histone H2A.X (Ser139) Antibody Sigma 05-636-I; Suitable for IHC/ICC of human and mouse. Validated for detecting nucleus of SPC in testis.
Rabbit monoclonal Anti-c-Jun antibody [E254] Abcam Ab32137; Suitable for WB, IHC/ICC of human . Validated for detecting nucleus of SPC in testis.
Mouse monoclonal Anti-Enolase Antibody (A-5) Santa Cruz sc-271384; Suitable for WB, IHC/ICC of human. Validated for detecting cytoplasm of SC in testis.
Rabbit Polyclonal Anti-DEFB119 Antibody Atlas Antibodies HPA043059; Suitable for IHC of human. Validated for detecting cytoplasm of SC in testis.
Mouse monoclonal Anti-GATA-4 Antibody (G-4) Santa Cruz sc-25310; Suitable for IHC of human. Validated for detecting nucleus of SC

in seminiferous tubules.
Rabbit monoclonal Anti-ZO-1 antibody (D6L1E) CST #13663; Suitable for WB, IHC/ICC of human and monkey. Validated for detecting tight junction in seminiferous tubules.
Mouse monoclonal Anti-EGR3 Antibody (A-7) Santa Cruz sc-390967; Suitable for WB, IHC/ICC of human. Validated for detecting nucleus of immature SC.
Rabbit Polyclonal Anti-HOPX Antibody Proteintech 11419; ISuitable for WB, IHC/ICC of human. Validated for detecting nucleus of mature SC.
Mouse monoclonal Anti-HOPX (E-1) Antibody Santa Cruz; sc-398703; Suitable for WB, IHC/ICC of human. Validated for detecting nucleus of mature SC.
Rabbit Polyclonal Anti-INHA Antibody Atlas Antibodies HPA019141; Suitable for IHC/ICC of human. Validated for detecting cytoplasm of SC.
Mouse monoclonal Human Activin RIIB Antibody R&D MAB3392; Suitable for WB and IHC of human. Validated for detecting membrane of SSC.
Mouse monoclonal Anti-TGFβ RIII (A4) Antibody Santa Cruz sc-74511; Suitable for WB and IHC of human. Validated for detecting membrane of LC.
Rabbit Polyclonal SOX9 Antibody millipore AB5535; Suitable for WB, ChIP, IHC/ICC of human, mouse, rat and chicken. Validated for detecting nucleus of SC.
Rabbit monoclonal [SP171] SMA Antibody Abcam ab150301.Suitable for Flow Cyt, WB, IHC/ICC of human, mouse and rat. Validated for detecting cytoskeleton of myoid cells.
Mouse monoclonal Anti-Active-β-Catenin (Anti-ABC) Antibody millipore 05-665. Suitable for Flow Cyt, WB, IHC of human, mouse and rat. Validated for detecting membrane and nucleus of SC.
Mouse monoclonal Anti-β-Catenin Antibody Servicebio GB12015. Suitable for WB and IHC/ICC of human, mouse and rat. Validated for detecting membrane and nucleus of SC.

SSC:spermatogonial stem cell; SPG: spermatogonia; SPC: spermatocyte; SPT: spermatid; SC: Sertoli cells; LC: Leydig cells

Eukaryotic cell lines

Policy information about cell lines

Cell line source(s)	Human primary Sertoli cells from OA and iNOA testis; mouse GS cell line
Authentication	The cells were identified by SOX9 (Sertoli cells) and Nanos2 (mouse SSCs) staining.
Mycoplasma contamination	All cell lines tested were negative for mycoplasma contamination.
Commonly misidentified lines (See ICLAC register)	No commonly misidentified cell lines were used.

Human research participants

Policy information about studies involving human research participants

Population characteristics	Total 17 male donors were recruited. They all belong to the Han Chinese. Ten normal samples (5 underage, including 2, 5, 8, 11, 17 years old and 5 Obstructive azoospermia donors age from 23 to 31yo) all had normal karyotypes, genotypes, sex hormone levels, and morphology of seminiferous tubules according to their age. The three KS patients (age from 26 to 29yo) and AZFa microdeletion donors (31yo) were diagnosed by spectral karyotyping and RT-PCR. The qPCR examination before hospitalization showed that in this sample, sY84 and sY86 were completely deleted. Among all donors, other abnormal genotypes related to spermatogenic disorders were excluded by whole-exome sequencing.
Recruitment	Patients (their parent if under-age) were asked if they wanted to join the study before admission. Fresh testicular tissues were obtained from 5 male donors who underwent testicular biopsy or partial excision for the following indications: contralateral testis to testicular torsion (17 yo patient; n = 1), benign testicle mass (2, 5, 11 years old patients; n = 3), or contralateral testis to cryptorchidism (8 yo patient; n = 1), and an additional 5 Obstructive azoospermia and 7 Non-obstructive azoospermia samples were obtained from the abandoned tissues after testicular sperm extraction operation. There may be racial bias due to the fact that the samples are from Han Chinese population, but this study also combined relevant research from another US laboratory, and the combination of the two data sets can eliminate this bias.
Ethics oversight	The experiments performed in this study were approved by the Ethics Committee of Shanghai General Hospital (License No. 2016KY196). For human testis samples, all participants (and their legal guardian if aged <18 years) signed their consent after being fully informed of the goal and characteristics of our study. The relevant informed consent is attached.

Note that full information on the approval of the study protocol must also be provided in the manuscript.
